# Determination of oxygen relaxivity in oxygen nanobubbles at 3 and 7 Tesla

**DOI:** 10.1007/s10334-022-01009-3

**Published:** 2022-04-13

**Authors:** Emma Bluemke, Liam A. J. Young, Joshua Owen, Sean Smart, Paul Kinchesh, Daniel P. Bulte, Eleanor Stride

**Affiliations:** 1 Department of Engineering Sciences, Institute of Biomedical Engineering, University of Oxford, Oxford, UK; 2 Radcliffe Department of Medicine, Oxford Centre for Clinical Magnetic Resonance Research, University of Oxford, Oxford, UK; 3 Clinical Center, National Institutes of Health, Bethesda, MD USA; 4 Department of Oncology, Radiobiology Research Institute, University of Oxford, Oxford, UK

**Keywords:** Quantitative MRI, MRI relaxometry, Oxygen, Hypoxia

## Abstract

**Objective:**

Oxygen-loaded nanobubbles have shown potential for reducing tumour hypoxia and improving treatment outcomes, however, it remains difficult to noninvasively measure the changes in partial pressure of oxygen (*P*O_2_) in vivo. The linear relationship between *P*O_2_ and longitudinal relaxation rate (*R*_1_) has been used to noninvasively infer *P*O_2_ in vitreous and cerebrospinal fluid, and therefore, this experiment aimed to investigate whether *R*_1_ is a suitable measurement to study oxygen delivery from such oxygen carriers.

**Methods:**

*T*_1_ mapping was used to measure *R*_1_ in phantoms containing nanobubbles with varied *P*O_2_ to measure the relaxivity of oxygen (*r*_1Ox_) in the phantoms at 7 and 3 T. These measurements were used to estimate the limit of detection (LOD) in two experimental settings: preclinical 7 T and clinical 3 T MRI.

**Results:**

The *r*_1Ox_ in the nanobubble solution was 0.00057 and 0.000235 s^−1^/mmHg, corresponding to a LOD of 111 and 103 mmHg with 95% confidence at 7 and 3 T, respectively.

**Conclusion:**

This suggests that *T*_1_ mapping could provide a noninvasive method of measuring a > 100 mmHg oxygen delivery from therapeutic nanobubbles.

**Supplementary Information:**

The online version contains supplementary material available at 10.1007/s10334-022-01009-3.

## Introduction

Tumour hypoxia is a predictor of disease progression, treatment failures, and metastatic potential in multiple types of cancer [1–4]. Strategies for treating hypoxia have included methods such as blood transfusions, hypoxia-selective drugs, and direct oxygen delivery [5]. The primary methods for oxygen delivery have generally focused on: hyperbaric oxygen or high oxygen content breathing therapy, oxygen-generating materials, and oxygen-carrying materials [6–8]. More recent approaches to hypoxia mitigation have taken advantage of developments in drug-delivery systems to deliver oxygen to the tumour. Of these drug-delivery approaches, therapeutic and diagnostic applications of nano- and micro-materials are playing increasingly important roles [9], such as micro and nanobubbles which can be used to encapsulate oxygen [10–16]. McEwan et al*.* [13] have shown impressive results using microbubble-delivered oxygen for improving sonodynamic therapy in pancreatic tumours, Eisenbrey et al*.* [17] have successfully increased breast tumour oxygenation levels in vitro by 20 mmHg, and Owen et al*.* [16, 18] have demonstrated that oxygen nanobubbles enhanced tumour response to sonodynamic therapy. These results have illustrated the potential of oxygen loaded nano- and microbubbles to deliver oxygen to the tumour microenvironment, reduce tumour hypoxia, and improve treatment outcomes [13, 16].

However, despite the enhanced therapeutic responses, statistically significant changes in tumour oxygenation were not always observed when this was directly measured [13, 17, 18]. This makes it extremely difficult to establish the underpinning mechanism(s) by which the micro/nanobubbles are promoting therapeutic effects. A key challenge has been that the methods available for measuring changes in oxygenation have multiple limitations. For example, Owen et al*.* [18] used a single fibre-optic probe and were only able to probe one spot in the tumour. Since tumours are highly heterogeneous in terms of perfusion, this resulted in a very high variability in the measurements. Alternatively, techniques may only be suitable for measuring either dissolved oxygen or oxygen bound to haemoglobin and thus not able to detect oxygen encapsulated within bubbles. For example, Eisenbrey et al*.* were unable to detect any change in oxygenation using photoacoustic imaging, but this is unsurprising since it relies on comparing ratios of oxygenated and deoxygenated haemoglobin, whereas the delivery of oxygen using micro- and nanobubbles is independent of haemoglobin transport [19]. Therefore, to continue effective research on promising oxygen carriers and hypoxia reduction, robust methods for measuring oxygen delivery from these carriers are needed.

The ideal method should be noninvasive and quantitative, allowing tumour oxygen measurements to be obtained before, during, and after treatment. As magnetic resonance imaging (MRI) is a widely clinically available, non-ionizing, noninvasive imaging technique, it is very well suited for determining tissue oxygenation. Molecular oxygen is paramagnetic [20–27], and it has been demonstrated experimentally repeatedly that the relaxation rate of the imaged material, *R*_1_, is linearly proportional to the concentration or partial pressure of oxygen (*P*O_2_) [21, 28–40]:

1 $${R}_{1\mathrm{Ox}}={R}_{10} +{r}_{1\mathrm{Ox}}\times C,$$

where *R*_1Ox_ is the relaxation rate with oxygen added, *R*_10_ is the relaxation rate without oxygen, *C* is the concentration or partial pressure of oxygen, and *r*_1Ox_ is the relaxivity of oxygen in the imaged material (whose units depend on the oxygen measurement used for *C*). Since the partial pressure of oxygen (*P*O_2_) is a common measurement in biomedicine and clinical applications, in this manuscript, we report *C* as *P*O_2_ in mmHg and *r*_1Ox_ as s^−1^/mmHg.

This linear, reproducible relationship between *P*O_2_ and *R*_1_ has been used to infer oxygen levels in vitreous fluid as a noninvasive alternative to the highly invasive oxygen electrodes used to measure retinal hypoxia [28, 30, 39, 41], bladder urine [41] and urine in the renal pelvis to create a noninvasive detection of renal dysfunction [42], cerebrospinal fluid [33, 41], brain tissue [43, 44], and blood, including foetal blood [36, 45, 46]. Therefore, we hypothesized that this method could also be applicable as a noninvasive method for measuring oxygen delivery from these nanocarriers.

However, changes in *R*_1_ are not specific to oxygen: the *R*_1_ of the material can be affected by the temperature [39, 41], pH [47], protein concentration [30], field strength [48], and numerous other changes in the chemical environment [49]. Therefore, unless the specific *P*O_2_–*R*_1_ relationship was measured in the material of interest previously (with consistent temperature, scanner protocol, and protein concentration), absolute oxygen levels cannot be inferred from *R*_10_ [41], only a relative change in oxygenation, since the *y*-intercept of the linear relationship is not known. The nanobubbles used for oxygen delivery in the studies discussed above are small lipid-based particles, ~ 100 nm in diameter, within an acidic (pH 2.3) solution. The relaxivity of oxygen in this nanobubble mixture has not yet been measured, however, analogous work by Thompson et al*.* demonstrated a measurable change in oxygenation in a phantom before and after destruction of oxygen-filled microbubbles [50]. Thompson et al*.* found that the linear relationship between *P*O_2_ and *R*_1_ in a phantom containing microbubble solutions of varying *P*O_2_ showed a relaxivity of 0.0003 s^−1^/mmHg at 7 T. With a similar objective, Vatnehol et al*.* measured the relaxivity and limit of detection of oxygen at 3 T in “oxygen-enriched” water intended for therapeutic oxygen delivery [37].

We have built upon their work in the experiments in this manuscript to investigate whether MRI *T*_1_ mapping is a suitable technique to examine oxygen delivery from such carriers. Therefore, we hypothesized that this method could also be applicable as a noninvasive method for measuring oxygen delivery from these nanocarriers and that the additional substances in the nanobubble solution would not interrupt the linear relationship between *R*_1_ and *P*O_2_ in the solution. To examine this, we performed two separate experiments to find the relaxivity and limit of detection of oxygen in this nanobubble mixture in two experimental scenarios: a preclinical 7 T MRI and clinical 3 T MRI.

## Methods

### Phantom design

For the 7 T experiment phantom, seven vials containing different solutions were prepared: oxygen-filled nanobubbles, nitrogen-filled nanobubbles, air-filled nanobubbles, water, oxygenated water, and a half oxygen-filled and half nitrogen-filled nanobubble mixture. One 0.5 ml syringe was filled per solution and sealed using Cristaseal wax (ProSciTech Pty Ltd, Australia). A seven-chamber custom-built holder was used to hold seven 0.5 ml syringes in the scanner for the duration of the phantom experiment.

For the 3 T experiment phantom, glass vials (10 ml) containing different solutions were prepared: five vials of air-filled nanobubbles, five vials of oxygen-filled nanobubbles, five vials of water, and five vials of water sparged with 100% oxygen. All nanobubble and water solutions were prepared according to the method listed in the section below. The vials were placed in a cylindrical vessel with saline solution surrounding them for the duration of the phantom experiment.

Lastly, to examine the effect that temperature may have on the experiment, three phantom tubes from a calibration phantom (Eurospin II TO5 phantom, Diagnostic Sonar LTD, Livingston, Scotland) were used in the scanner and a Variable Flip Angle (VFA) *T*_1_ map acquired five times, while monitoring the temperature. The three phantom tubes had nominal *T*_1_ values of 830, 1020, and 1350 ms.

### Nanobubble preparation

All nanobubble solutions were prepared according to the following method: lecithin and citric acid were obtained from Special Ingredients (Chesterfield, Derbyshire, UK). Glycyrrhizic acid and glycerol were obtained from Sigma-Aldrich Ltd (Gillingham, Dorset, UK). Oxygen and nitrogen cylinders were obtained from BOC gases (Guildford, Surrey, UK). The nanobubble solutions were prepared according to Owen et al*.* (2016). To create one 100 ml bottle of the solution, glycyrrhizic acid (0.5 mg/ml), lecithin (3 mg/ml), citric acid (5 mg/ml) and glycerol (0.0125 ml/ml) were mixed with 100 ml of freshly boiled, filtered deionized water. The solution was then stirred for 30 min, while it cooled to room temperature. The vial was then immediately sealed and mechanically agitated for 30 s. To produce oxygen-laden nanobubbles, 5 ml of the solution was transferred to a glass vial and sparged with oxygen gas, for 3 min. To produce nitrogen or air-laden nanobubbles, the solution was sparged with nitrogen gas or air for 3 min. The nanobubble size distribution measurements were ascertained using a nanoparticle tracking analyzer (NTA) (Nanosight NS300, Malvern, PA) and all size distributions were consistent with results published by Owen et al. [16].

### MRI acquisition details

The 7 T imaging experiments were performed using a 7.0 T 210 mm horizontal bore VNMRS preclinical imaging system equipped with 120 mm bore gradient insert (Varian Inc. Palo Alto, CA, USA). A variable flip-angle *T*_1_ map [51] was calculated using non-linear least squares from 3D RF and gradient spoiled gradient-echo sequence with 16 flip angles (TR = 3.2 ms, TE = 0.664 ms, matrix = 128 × 64 × 64, FA = 1, 1.2, 1.4, 1.6, 1.8, 2, 2.2, 2.4, 2.7, 3.1, 3.7, 4.4, 5.2, 6.1, 7, 8, FOV = 54 × 27 × 27 mm^3^), and B1 correction was applied using Actual Flip Angle method [52]. The 16 flip angles were chosen according to the Ernst angle for a large range of values of tissue *T*_1_—using more flip angles allows for a more accurate fit for the calculation of *T*_1_. *T*_1_ values were measured and averaged in a region of interest (ROI) placed lengthwise along each syringe to produce a mean *T*_1_ value for each syringe.

The 3 T imaging experiments were performed using a 3.0 T Siemens Prisma MRI (Erlangen, Germany). The VFA *T*_1_ maps were calculated from a linear fit of [51] 3D gradient-echo images with five different flip angles (TR = 4.1 ms, TE = 1.23 ms, FA = 3, 6, 9, 12, 15, slice thickness = 3 mm). The Modified Look-Locker Inversion Recovery (MOLLI) [53] and Shortened Modified Look-Locker Inversion Recovery (ShMOLLI) *T*_1_ maps were calculated from a non-linear fit using a MOLLI *T*_1_-mapping sequence (TR = 3.5 ms, TE = 1.05 ms, 11 inversion times, FA = 35, slice thickness = 8 mm), and Shortened Modified Look-Locker Inversion Recovery (ShMOLLI with the acquisition details: (ShMOLLI_192i protocol, TR = 371.84 ms, TE = 1.01 s, FA = 35, slice thickness = 8 mm, 7 inversion times TI = 100, 1100, 2100, 3100, 4100, 180, 260 ms). Each type of *T*_1_ map was acquired multiple times, repeated in the following order: ShMOLLI 1, MOLLI 1, VFA 1, ShMOLLI 2, MOLLI 2, VFA 2, ShMOLLI 3, MOLLI 3. The ShMOLLI *T*_1_-mapping method produced the lowest standard deviation within each vial ROI and was, therefore, used for the relaxivity and limit of detection (LOD) calculations (see Supplementary Figure S1). Although multiple *T*_1_ maps were acquired for this experiment, the ShMOLLI *T*_1_ map acquired closest to the time point of the collection of the oxygen measurement (ShMOLLI 3) was used to calculate the following relaxivity and LOD results. This decision was made for two reasons: (1) the ShMOLLI 3 and MOLLI 3 data was acquired closest to the time the oxygen measurements were made, and thus provide the *T*_1_ measurement most closely corresponding to the measured *P*O_2_ if oxygen levels were leaking or decreasing over time; and (2) the ShMOLLI *T*_1_ map was chosen over the MOLLI *T*_1_ map due to an artefact corruption on the MOLLI *T*_1_ map. The *T*_1_ measurement from each repetition of the 3 ShMOLLI *T*_1_ maps was compared in Supplementary Figure S2.

The resulting *T*_1_ maps used for analysis are shown in Supplementary Figure S3. Note that the scale bars highlights the size differences between the phantoms, and therefore the field of view shown in each image: the 7 T phantom used small 0.5-ml syringe vials to fit into a small-bore preclinical scanner, while the 3 T phantom used much larger 10 ml glass vials within a large saline bucket. The resolution of the ShMOLLI image is 1.7 mm × 1.7 mm, and the resolution for the VFA slice displayed was 0.5 mm × 0.5 mm.

### Oxygen measurements

Following the 7 T scans, the remaining solution in each of the seven larger vials was measured using a fibre-optic oxygen sensor. All *P*O_2_ measurements were carried out using a PreSens OXY Mini-fibre-optic oxygen meter in combination with SP-PSt3-NAU oxygen sensor spots inside sealed glass vials (PreSens Precision Sensing GmbH, Regensburg, Germany). All oxygen measurements were calibrated and carried out at room temperature according to PreSens manual with oxygen-free water and air-saturated water, as performed by Owen et al. [16, 18].

Following the 3 T scans, the phantom was carefully removed from the scanner, and one by one, each vial was quickly opened and poured into a vial with a PreSens sensor sticker with the PreSens tip recording the *P*O_2_ measurement (PreSens OXY Mini fibre-optic oxygen meter). The *P*O_2_ was recorded for 30 s, recording one measurement per second. All oxygen measurements were calibrated and carried out at room temperature. The oxygenated samples were recorded first, then the non-oxygenated samples, to reduce the time over which the oxygen levels could be decreasing due to exchange with the room air.

### Calculation of limit of detection

The limit of detection of oxygen in the solutions can be calculated by the following equation [54]:

2 $$\mathrm{LOD}=\frac{F\times \mathrm{SD}}{b},$$

where *b* is the slope of the regression line (relaxivity, or *r*_1Ox_), SD is the standard deviation of the intended in vivo situation, and *F* is a factor set to 2.2 for LOD calculations with 95% confidence intervals. This method is used by Vatnehol et al. to calculate the limit of detection of dissolved oxygen in water [37].

## Results

### Oxygen relaxivity

**Fig. 1:**
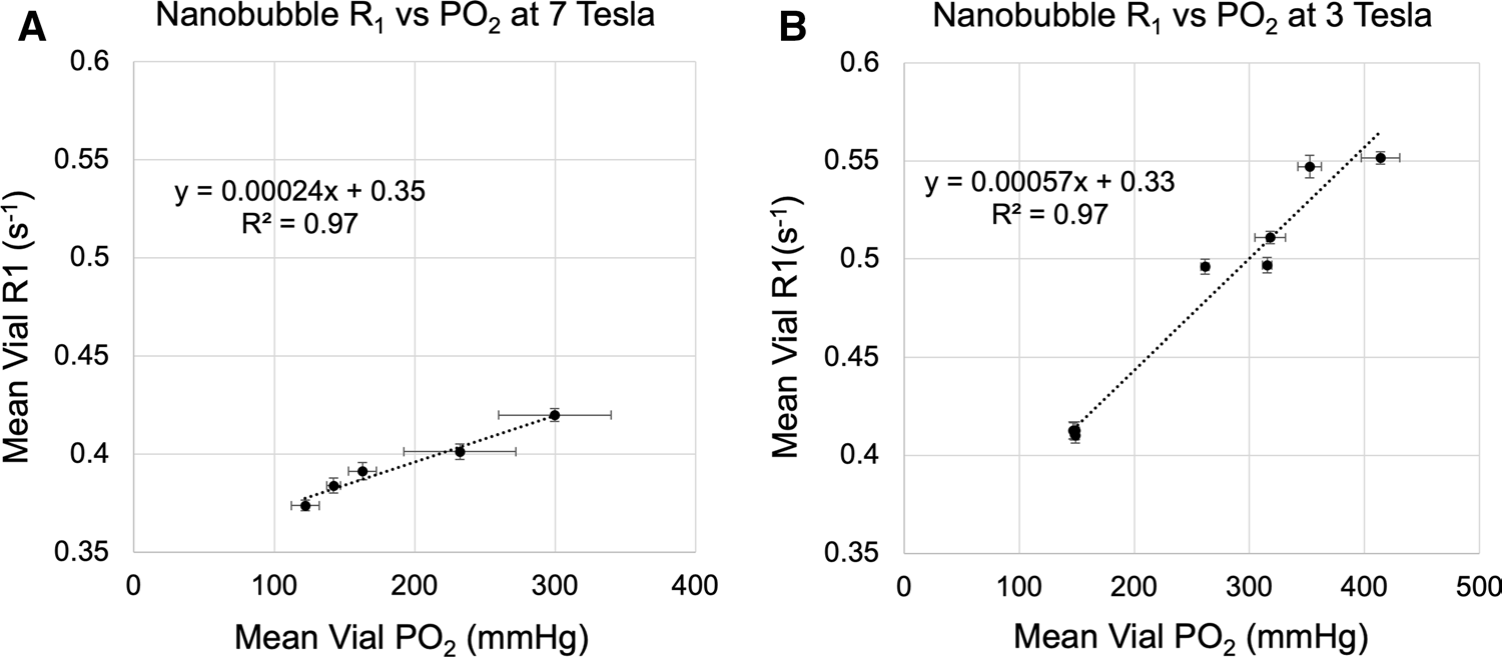
The mean *R*_1_ values (s^−1^) and mean *P*O_2_ values (mmHg) in the nanobubble solutions at 7 T and 3 T, plotted with a linear regression line (*R*^2^ = .97 and *R*^2^ = .97) and relaxivity slope (*r*_1Ox_) of 0.000235 s^−1^/mmHg and 0.00057 s^−1^/mmHg, respectively. The corresponding upper and lower confidence intervals and *P* values are listed in Table 1. To view this figure with cropped axes, see Supplementary Figure S6

The phantoms and resulting *T*_1_ maps can be seen in Supplementary Figure S3. We measured a strong linear relationship (*R*^2^ = 0.97 and *R*^2^ = 0.97) between *P*O_2_ and *R*_1_ in the nanobubble solution in vitro at 7 and 3 T, respectively, shown in Fig. 1. The relaxivity (*r*_1Ox_) at 7 and 3 T was 0.00024 s^−1^/mmHg and 0.00057 s^−1^/mmHg, respectively, and the corresponding upper and lower confidence intervals and P values are listed in Table 1.

**Table 1:** The resulting *R*^2^, *P* value and slope with lower and upper 95% confidence intervals and for each linear regression shown in Fig. 1

Data	Slope (95% CI) [units]	*P* value	*R*^2^
*R*_1_ vs *P*O_2_ at 7 T (Fig. 2A)	0.00024 (0.00016, 0.00031) [s^−1^/mmHg]	< 0.0001	0.97
*R*_1_ vs *P*O_2_ at 3 T (Fig. 2B)	0.00057 (0.00048, 0.00065) [s^−1^/mmHg]	0.00016	0.97

### Limit of detection

The SD in the LOD equation represents the standard deviation of the method in the intended tissue. Therefore, for the 7 T preclinical setting, the mean of eight reported standard deviation values of *R*_1_ from the intended experimental tissue—a preclinical tumour model—was calculated, which was approximately 0.011 s^−1^ [55]. Using the measured relaxivity at 7 T (0.000235 s^−1^/mmHg) and Eq. 1, the resulting LOD (95% confidence) at 7 T in a preclinical tumour model is 103 mmHg. For the 3 T clinical setting, the mean of 11 reported standard deviation values of *R*_1_ from clinical tumour ROI was calculated, which was approximately 0.029 s^−1^ [56]. Using the measured relaxivity at 3 T (0.00057 s^−1^/mmHg) and Eq. 1, the resulting LOD (95% confidence) at 3 T in a clinical tumour is 111 mmHg. Of course, it is likely that measurements from *T*_1_ mapping methods will be less precise in vivo than in phantoms—therefore, in vivo measurements for SD were used in these LOD calculations.

### Nanobubbles and R_1_

**Fig. 2:**
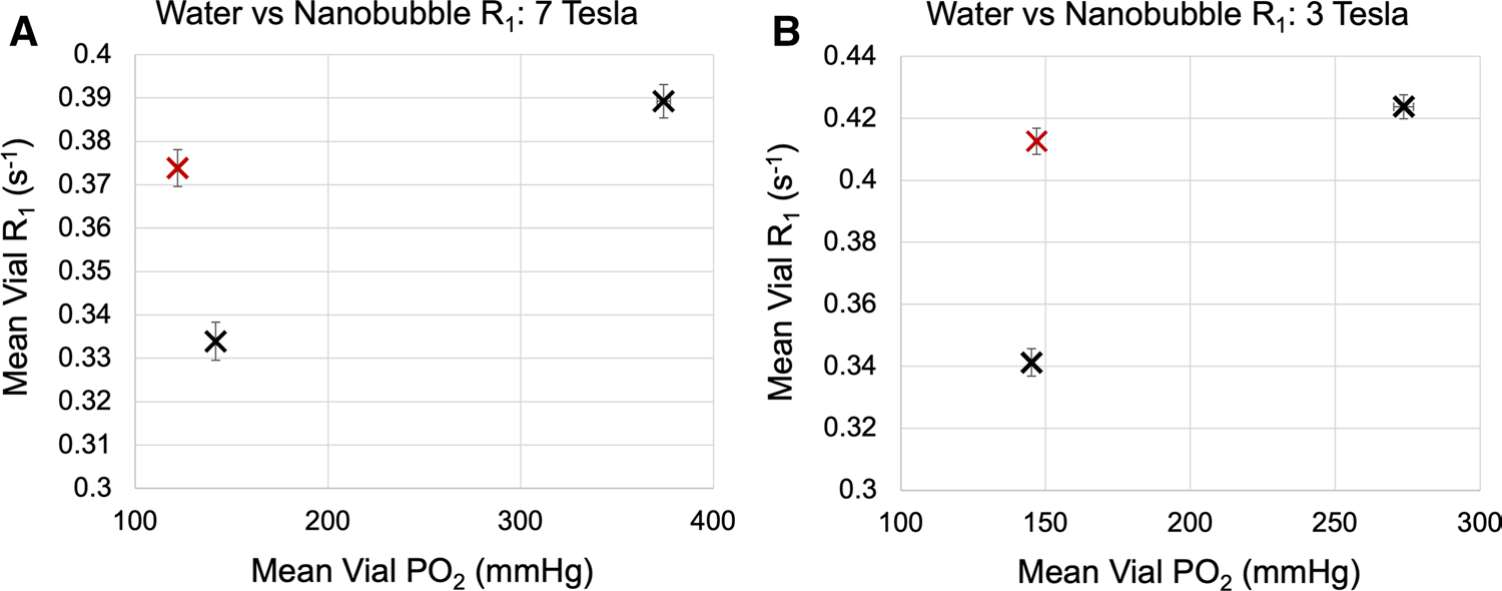
The mean *R*_1_ and *P*O_2_ values of the vials of water and oxygenated water (black crosses), in comparison to non-oxygenated nanobubbles (red cross), at **A** 7 T and **B** 3 T. The contents of the non-oxygenated nanobubble solution resulted in an *R*_1_ that was 0.03 and 0.07 s^−1^ higher than water, the equivalent effect as increasing the water *P*O_2_ by 230 and 130 mmHg (at 7 and 3 T, respectively)

In comparison to water, the nanobubble solution contains several attributes that may affect *R*_1_, in particular, the presence of the lipid-based particles, and the acidity (pH 2.3) of the solution. It was apparent from both the 7 and 3 T experiments (Fig. 2) that the nanobubble solution alone—*without* oxygen added—produced an increase in *R*_1_ of 0.03 and 0.07 s^−1^ compared to water; equivalent to increasing the water *P*O_2_ by 230 and 130 mmHg (at 7 and 3 T, respectively).

The experiment measuring the effect of temperature on *R*_1_ showed that the *R*_1_ of all three tubes decreased as temperature increased, and a linear regression fit strongly (*R*^2^ = 0.83, 0.90, 0.91). The change in *R*_1_ per change in temperature was − 0.037, − 0.027, and − 0.020 s^−1^/°C for the three tubes, respectively (see Supplementary Figure S4), and a steeper slope corresponded to a higher baseline *R*_1_, and vice versa. The corresponding upper and lower confidence intervals and P values are listed in Supplementary Table S1. This means that from just a 0.5 °C fluctuation in temperature, in *R*_1_ would change by 0.018 s^−1^, which would correspond to a 32 and 77 mmHg inaccuracy in *P*O_2_ estimation, at 3 and 7 T, respectively. The same data are also provided in terms of *T*_1_ in Supplementary Figure S5, where a linear regression fit *T*_1_ vs temperature data strongly as well (*R*^2^ = 0.83, 0.90, 0.91) with a slope of 20, 23, and 29 ms/°C for the three tubes, respectively. These results are consistent with findings by Zhang et al*.* where the temperature sensitivity increased with baseline *T*_1_, following closely a second-order polynomial (see Fig. 2 in Zhang et al.) [57].

## Discussion

The relationship between *P*O_2_ and *R*_1_ has been used to noninvasively infer oxygen levels in vitreous fluid [28, 30, 39, 41], urine [41, 42], cerebrospinal fluid [33, 41], brain tissue [43, 44], and blood [36, 45, 46]. Therefore, we hypothesized that this method could also be applicable as a noninvasive method for measuring oxygen delivery from these nanocarriers. To examine this, we performed two separate experiments to find the relaxivity and limit of detection of oxygen in this nanobubble mixture in two experimental scenarios: a preclinical 7 T MRI and clinical 3 T MRI.

### Relaxivity

These experiments reproduced the linear *R*_1_–*P*O_2_ relationship expected (*R*^2^ = 0.97), and showed that the *P*O_2_ of the nanobubble solution could be estimated using *T*_1_ mapping at 7 and 3 T. It was a possibility that the composition of the nanobubble solution would interrupt this linear *R*_1_–*P*O_2_ relationship or induce image artefacts. Fortunately, this was not found to be the case.

The *r*_1Ox_ in the nanobubble solution was 0.00024 and 0.00057 s^−1^/mmHg at 7 and 3 T respectively. In these experiments, the *r*_1Ox_ was stronger at the lower field strength, which agrees with other reports from phantom measurements of *r*_1Ox_ being greater at lower field strengths [58–60]. The comparable experiments by Thompson et al*.* [50] found an *r*_1Ox_ of 0.0003 s^−1^/mmHg at 7 T, which within the upper 95% confidence interval of our measurement performed at 7 T (0.00031 s^−1^/mmHg). Therefore, even though the composition of the microbubble solution and nanobubble solutions differ considerably (see Thompson et al*.* [50] for the microbubble formula), the *r*_1Ox_ seems to be almost independent of the nano or microbubble composition, which is a notable strength of this method.

### Limit of detection

Using our measured *r*_1Ox_ and published values of the expected standard deviation in *R*_1_ within preclinical and clinical tumour tissue, the resulting 95% confidence interval LOD calculated indicates that a 103 mmHg (4.56 mg/L) and 111 mmHg (or 7.04 mg/L) increase in oxygen is required to reliably detect a change in *R*_1_ from oxygen delivery (in a preclinical and clinical setting, respectively). These are both slightly lower than the 8.5–12.3 mg/L limit of detection estimated by Vatnehol et al., however, for their calculations, the authors use the standard deviation in the hepatic portal vein as that is their material of interest [37]. Of course, this raises the most important point of this discussion: these LOD calculations assume that there are no other potential *T*_1_ changing effects present other than an increase in oxygen—which, in the body, is an often unrealistic assumption, especially in a voxel containing blood. The other main endogenous paramagnetic material in the body is deoxyhemoglobin, and the *R*_1_ of blood decreases linearly as oxygen saturation increases (i.e. as deoxyhemoglobin is converted to oxyhemoglobin, which is diamagnetic) [34, 61, 62]. Only following full oxygen saturation (*S*O_2_ > 99%) will the *R*_1_ of blood increase linearly due to the increase in the *P*O_2_ of the plasma being the dominant remaining effect [29, 34, 36, 45, 62]. Therefore, if an oxygen delivery method results in an increase in oxygen saturation of the blood, it is likely to induce a *negative* change in *R*_1_ first, until it delivers enough oxygen to reach full saturation [62]. In fact, this is precisely what was observed by Vatnehol et al*.* when they performed their intended oxygen delivery experiment in humans and measured a *negative* *R*_1_ change in the blood [63]. However, for a voxel that does not contain 100% blood, such as normal tissue, it has been demonstrated that the *R*_1_ of the tissue will increase linearly with the level of dissolved oxygen in the tissue [43]. Since the effect from changing deoxyhemoglobin levels will only pertain to the percent volume in the voxel that is occupied by blood, then the remaining 90–98% of voxel volume will be dominated by *R*_1_ change of the tissue. In addition to deoxyhemoglobin, iron levels present in the tissue would affect *R*_1_ as well, and it can accumulate depending on the disease and organ. Therefore, while the LOD calculations in this manuscript do not account for confounding effects on *R*_1_, they are still useful to obtain an estimation of whether the detection of nanobubble oxygen delivery by *R*_1_ measurement is feasible.

The expected *P*O_2_ change in the tumour tissue following the administration of nanobubbles has been reported to be approximately 30 mmHg, albeit this was measured using a method that was likely underestimating the *P*O_2_ [16]. This is much lower than both the LODs calculated in this experiment (103 and 111 mmHg), however this LOD calculation is stating that the two peaks of the distributions are separated by 2.2 standard deviations, which is considerable. Therefore, although the expected oxygen delivery is below the measured LOD, this could still be a feasible method for detecting the expected change in *P*O_2_. In addition, this measurement describes the LOD for a single voxel, which is different from the detection power in a large ROI—due to the repeated number of voxel measures in a large ROI, it would be feasible to detect a much smaller difference between two large ROIs that would otherwise not be realistic in a single voxel. Furthermore, the limit of detection will depend not only on the tumour type, and in particular its *T*_1_ values at a particular magnetic field, but also the site of the tumour—such as the head and neck region versus the pelvic region—which will affect the SNR characteristics, and may be a caveat to this estimate.

In addition, the standard deviation within the tumour ROIs used is a combination of both tissue heterogeneity and *T*_1_ mapping measurement variability, and as *T*_1_ mapping methods continue to improve, it is possible that the standard deviations could decrease significantly and oxygen detection could be made on a voxel-wise level. Therefore, although the resulting LODs calculated from this experiment are greater than the expected *P*O_2_ change, it is possible that as *T*_1_ mapping techniques become more stable, assessment of spatial variation of *P*O_2_ may then be possible compared to region averaging which is needed currently, enabling *T*_1_ mapping to be a suitable technique to examine oxygen delivery from these nanobubbles.

Lastly, the LOD measurement describes the sensitivity of this technique to detecting *P*O_2_ changes in an individual. As a paired measurement in a study across multiple subjects at 3 T, however, assuming that a similar standard deviation as previous clinical *R*_1_ measurements [56], then a study of 10, 13, or 15 participants would yield 80%, 90%, or 95% power, respectively (alpha 0.05) [64].

### Additional confounding effects

In comparison to water, the increased lipid content and acidity of the non-oxygenated nanobubble solution resulted in an *R*_1_ that was higher than water. The magnitude of this effect was the equivalent of increasing the water *P*O_2_ by 230 and 130 mmHg (at 7 and 3 T, respectively). Thus, it is likely that increasing the nanobubble concentration in a water or saline phantom solution would increase *R*_1_, making it difficult to use that change in signal to infer a change in *P*O_2_. This problem is similar to that reported by Vatnehol et al*.* [63], where the delivery of a dissolved oxygen water drink caused a dilution that changed *R*_1_ in both the control drink and the oxygen drink. It is possible that this dilution would be a negligible effect from the nanobubbles once spread out in the circulatory system, however, it could make oxygen nanobubble delivery indistinguishable from non-oxygenated nanobubble delivery. Since the percent volume of nanobubble solution would be far below 10% of the blood volume and the tissue voxel would contain below 10% blood volume, and since the low pH would be neutralized in the bloodstream, it is reasonable to conclude that this confound would not be as substantial as the *R*_1_ change from the *P*O_2_ change within the tissue dominating 85–98% of the voxel volume, assuming a range of fractional blood volume in tumours of approximately 2–15% [65].

There are additional remaining factors that can affect *R*_1_. Experiments using *R*_1_ to measure *P*O_2_ in vitreous fluid have demonstrated the strong effect of temperature on the *R*_1_ measurement [39, 41]. Unfortunately, this is not insignificant—Zaharchuk et al*.* reported that based on their phantom experiments, they found a potential error ± 19 mmHg for physiologic temperature fluctuations of ± 1 °C [41]. We believe this drift in *R*_1_ values shown in Supplementary Figure S2 is due to a combination of two factors: (1) the temperature of the vials increasing due to absorption of heat from RF field exposure, and (2) a slight decrease in *P*O_2_ in the vials as oxygen may be able to slowly leak out of the vial during the duration of the scanning session. Both of these effects independently would cause a decrease in *R*_1_. This possible decrease in oxygen throughout the scanning session is why ShMOLLI 3, the ShMOLLI acquired closest to the time of oxygen measurement, was used for the analysis.

### Experiment limitations

The gold-standard *T*_1_ mapping method to use would have been inversion recovery, since Look-Locker techniques such as ShMOLLI are known to underestimate the true *T*_1_ and VFA will overestimate the true *T*_1_ (seen in Supplementary Figure S1) [66]. However, these experiments were intended as preliminary work for preclinical and clinical experiments, and therefore, it was more appropriate to use the actual experimental set up and scanning techniques that could be used in mice and human volunteers and/or patients. In comparison to phantoms, scanning live subjects introduces issues such as motion from breathing and limitations to scan time, and therefore the faster VFA method and much faster ShMOLLI method were more appropriate than inversion recovery. Lastly, although the *T*_1_ might be over- or under-estimated, the *relative* change in *R*_1_ (and therefore *r*_1Ox_) should remain the same in each method.

In addition, the 95% confidence interval of the fit of the slope of the measurements was ± 30% at 7 T, and ± 16% at 3 T, which at first glance, suggests that the *T*_1_ measurements from the 7 T scans were less reliable. However, upon further examination of the underlying data, we believe this discrepancy is due to the oxygen measurements rather than the *T*_1_ measurements. For the two data points with the highest *P*O_2_ in the 7 T data, the *P*O_2_ measurements have larger error bars—this is due to those glass vials showing a larger range of *P*O_2_ during the PreSens measurement time due to being disturbed while being moved from the scanner. Therefore, it is possible that the true *P*O_2_ during the time of *T*_1_ measurement was slightly different than it is showing there, which would result in a slightly tighter alignment of the data points and more narrow CI range. However, the *T*_1_ measurements from the 7 T data were of relatively lower quality than *T*_1_ measurements from the 3 T data, which remains a limitation of this study.

A final noteworthy observation is that adjusting the concentration of nanobubble solution in each vial would also alter the *R*_1_, as shown by the difference between the ‘water’ vial and ‘nanobubble’ vial in Fig. 2. We did not perform measurements at multiple concentrations of nanobubbles, so we are unable to provide additional data on this, but we hypothesise that *R*_1_ would decrease change linearly with increasing nanobubble concentration. To clarify, in this experiment, any solution containing nanobubble solution contained the same concentration of nanobubble solution, and the oxygen levels were altered via the gas used, not by the nanobubble concentration.

### Conclusion

We measured a strong linear relationship (*R*^2^ = 0.97) between *P*O_2_ and *R*_1_ in the nanobubble solution, and measured the relaxivity of oxygen in the nanobubble solution to be 0.00057 s^−1^/mmHg and 0.000235 s^−1^/mmHg, and established that a 111 and 103 mmHg increase in oxygen is required to detect a change in *R*_1_ with 95% confidence at 7 and 3 T, respectively. This suggests that *T*_1_ mapping could provide a noninvasive method of measuring a > 100 mmHg oxygen delivery from oxygenated nanobubbles in therapy.

## Supplementary Information

Below is the link to the electronic supplementary material.

Supplementary file1 (DOCX 976 KB)

## Abbreviations

LOD: Limit of detection
MOLLI: Modified look-locker inversion recovery
*P*O_2_: Partial pressure of oxygen
*r*_1Ox_: Relaxivity of oxygen
*R*_1_: Longitudinal relaxation rate
ShMOLLI: Shortened modified look-locker inversion recovery
*S*O_2_: Blood oxygen saturation
*T*_1_: Longitudinal relaxation time
VFA: Variable flip angle
