# Supplementary material for: Determination of oxygen relaxivity in oxygen nanobubbles at 3 and 7 Tesla
Source: MAGMA. 2022 Apr 13;35(5):817–26. doi: 10.1007/s10334-022-01009-3 (PMC9463275; doi:10.1007/s10334-022-01009-3)
Supplement: Supplementary file 1 — Supplementary file1 (DOCX 976 KB) [file 10334_2022_1009_MOESM1_ESM.docx]

# Supplementary Material


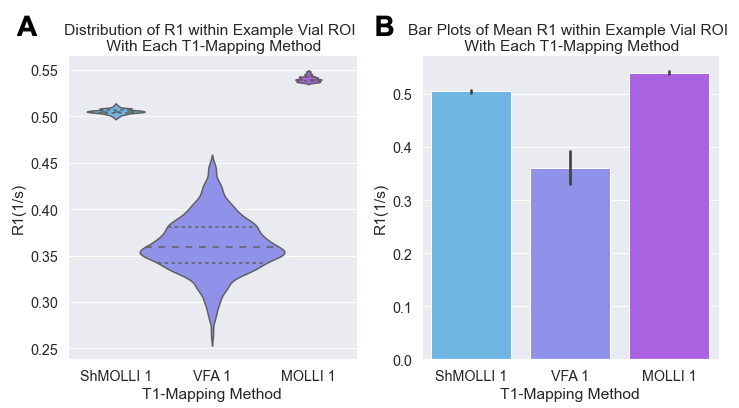


##### Supplementary Figure S1: (A) Violin plots of the distribution and (B) bar plots of the R_1_ within a representative example vial ROI collected from each T_1_ mapping method (Mean±SD): ShMOLLI (0.50±0.0027), VFA (0.36±0.031), and MOLLI (0.54±0.0031). The ShMOLLI T_1_-maps resulted in the lowest standard deviation and normal distribution within the ROIs and were therefore used for the relaxivity and LOD calculations. The vial contained the oxygenated nanobubble solution.


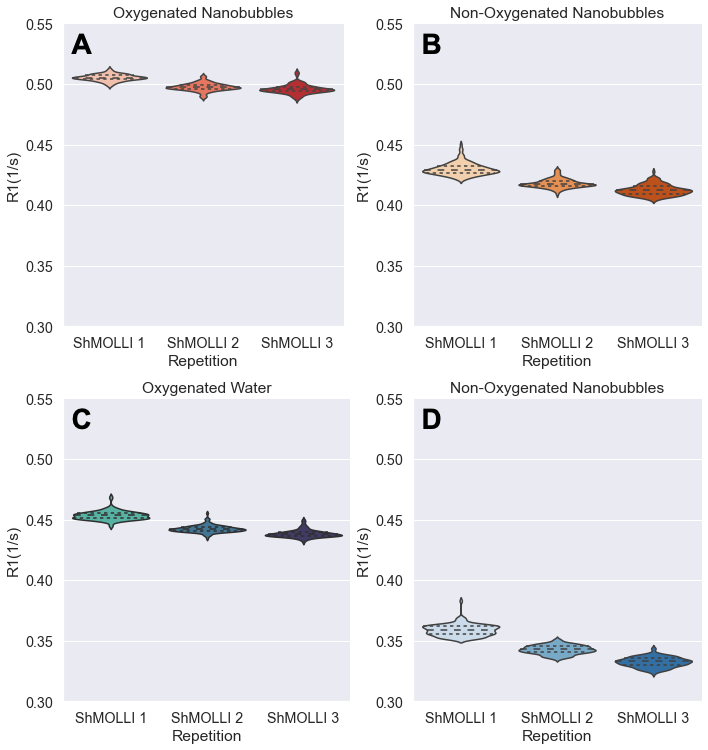
**Supplementary Figure S2:** Violin plots of the distribution of the R_1_ within a representative example vial ROI collected from each repetition of the 3 ShMOLLI T_1_-maps in (A) oxygenated nanobubbles, (B) non-oxygenated nanobubbles, (C) oxygenated water, and (D) non-oxygenated nanobubbles. Each y-axis has been cropped to ensure the distributions are visible, and each y-axis range encompasses 0.10s^-1^ in total to ensure each plot maintains the same scale.

#####

#####

#####
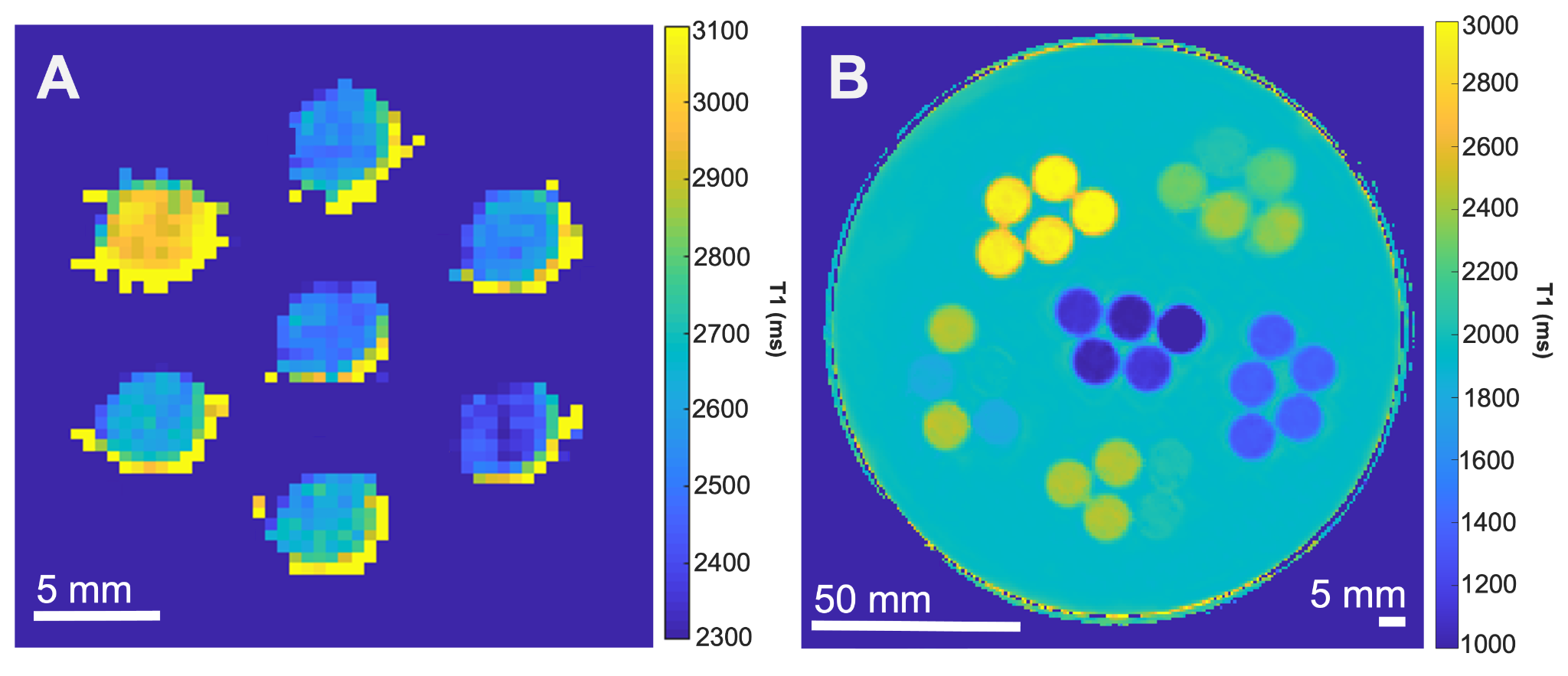


##### Supplementary Figure S3: Examples of the resulting T_1_ maps through a section of the vials in the (A) 7T and (B) 3T phantom. The scale bars illustrate the size differences between the phantoms: the 7T phantom used small 0.5 ml syringe vials to fit into a small-bore preclinical scanner, while the 3T phantom used much larger 10 ml glass vials within a large saline bucket. The resolution of the ShMOLLI image is 1.7 mm x 1.7 mm, and the resolution for the VFA slice displayed was 0.5 mm x 0.5 mm. The high T1 values at the edges of the 7T images are susceptibility artefacts from the interface between the liquid-filled glass syringes surrounded by air and a plastic syringe holder — the ROIs did not include those rim regions. These artefacts do not appear in the 3T vials due to being surrounded by saline fluid.

#####


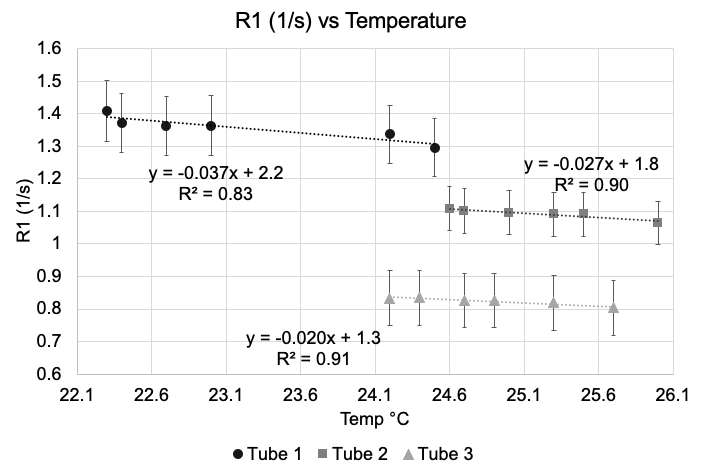


##### Supplementary Figure S4: To examine the effect that temperature may have on the experiment, VFA T_1_ maps of 3 calibration phantom tubes were repeated 5 times while monitoring the temperature. The R1 of all 3 phantom tubes (with a T_1_ of 830, 1020, and 1350 ms) decreased as temperature increased, and a linear regression fit strongly (R^2^=.83, .90, .91) with a slope of -0.037, -0.027, and -0.020 s^-1^/°C for the 3 tubes respectively. The corresponding upper and lower confidence intervals and P-values are listed in Supplementary Table S1.


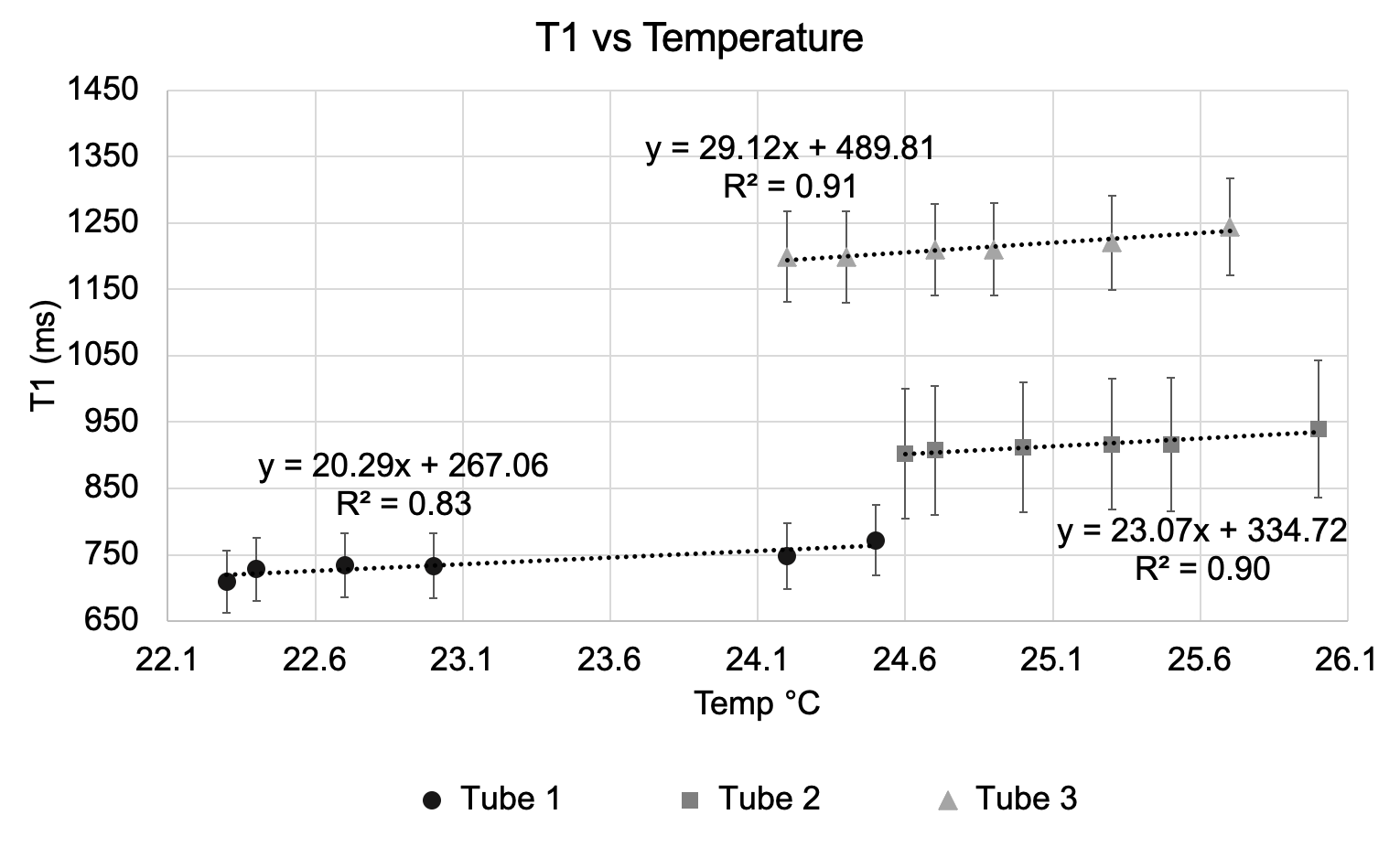


##### Supplementary Figure S5: The T_1_ of all 3 phantom tubes (with a T_1_ of 830, 1020, and 1350 ms) increased as temperature increased, and a linear regression fit strongly (R^2^=.83, .90, .91) with a slope of 20, 23, and 29 ms/°C for the 3 tubes respectively. The corresponding upper and lower confidence intervals and P-values are listed in Supplementary Table S1.

**
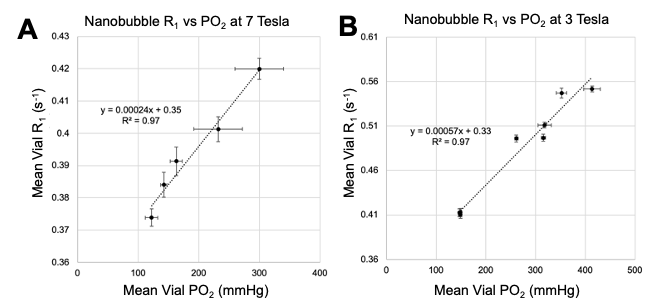
**

##### Supplementary Figure S6: The data from Figure 1, shown with axes cropped. The mean R_1_ values (s^-1^) and mean PO_2_ values (mmHg) in the nanobubble solutions at 7T and 3T, plotted with a linear regression line (R^2^=.97 and R^2^=.97) and relaxivity slope (r_1Ox_) of 0.000235 s^-1^/mmHg and 0.00057 s^-1^/mmHg, respectively. The corresponding upper and lower confidence intervals and P-values are listed in Table 1.

#####

#####

#####

##### Supplementary Table S1: The resulting R^2^, P-value and slope with lower and upper 95% confidence intervals and for each linear regression shown in Supplementary Figures S4 and S5.

| **Data** | | **Slope**  **(95% C.I.) [units]** | **P-value** | **R^2^** |
| --- | --- | --- | --- | --- |
| R_1_ vs Temp  (Fig S4) | Tube 1 | -0.036 (-0.060, -0.013) [s^-1^/°C] | 0.011 | 0.83 |
|  | Tube 2 | -0.027 (-0.040, -0.015) [s^-1^/°C] | 0.003 | 0.90 |
|  | Tube 3 | -0.019 (-0.027, -0.011) [s^-1^/°C] | 0.002 | 0.91 |
| T_1_ vs Temp  (Fig S5) | Tube 1 | 20.29 (7.74, 32.84) [ms/°C] | 0.011 | 0.83 |
|  | Tube 2 | 23.07 (12.22, 33.91) [ms/°C] | 0.004 | 0.90 |
|  | Tube 3 | 29.19 (16.36, 41.87) [ms/°C] | 0.003 | 0.91 |
